# Binding modes of CYP106A2 redox partners determine differences in progesterone hydroxylation product patterns

**DOI:** 10.1038/s42003-018-0104-9

**Published:** 2018-07-30

**Authors:** Tanja Sagadin, Jan L. Riehm, Mohammed Milhim, Michael C. Hutter, Rita Bernhardt

**Affiliations:** 1 Institute of Biochemistry, Campus B2.2, 66123 Saarbrücken, Germany; 2 Center for Bioinformatics, Campus E2. 1, 66123 Saarbrücken, Germany

## Abstract

Natural redox partners of bacterial cytochrome P450s (P450s) are mostly unknown. Therefore, substrate conversions are performed with heterologous redox partners; in the case of CYP106A2 from *Bacillus megaterium* ATCC 13368, bovine adrenodoxin (Adx) and adrenodoxin reductase (AdR). Our aim was to optimize the redox system for CYP106A2 for improved product formation by testing 11 different combinations of redox partners. We found that electron transfer protein 1(516–618) showed the highest yield of the main product, 15β-hydroxyprogesterone, and, furthermore, produced a reduced amount of unwanted polyhydroxylated side products. Molecular protein–protein docking indicated that this is caused by subtle structural changes leading to alternative binding modes of both redox enzymes. Stopped-flow measurements analyzing the CYP106A2 reduction and showing substantial differences in the apparent rate constants supported this conclusion. The study provides for the first time to our knowledge rational explanations for differences in product patterns of a cytochrome P450 caused by difference in the binding mode of the redox partners.

## Abstract (web summary)

Tanja Sagadin et al. show that different redox systems can be used to tune the rate selectivity and yield of progesterone conversion by the cytochrome P450 CYP106A2. They screen 11 redox partner combinations and identify specific combinations that may be used to improve biotechnological production of mono- and polyhydroxylated products.

## Introduction

CYP106A2 is a bacterial cytochrome P450 (P450) from *Bacillus megaterium* ATCC 13368 that is able to use a wide range of biotechnologically important substances as substrates, for example, steroids and terpenes^1–10^. Like almost all P450s, it requires electron transfer proteins to transmit electrons from NAD(P)H to the substrate^11^. A redox system as part of the 15β-hydroxylase system from *B. megaterium*, consisting of a NADPH-dependent flavoprotein and an iron-sulfur protein (designated as megaredoxin reductase and megaredoxin, respectively), has been purified and shown to provide the CYP106A2 with electrons, but has never been cloned^2,12^. It was later reported that CYP106A2 can also accept electrons from the adrenal redox system, comprising adrenodoxin (Adx) and adrenodoxin reductase (AdR)^13,14^. More research finally led to the use of the truncated version of Adx, Adx(4–108)^15,16^. Since genome mining of the *B. megaterium* strain DSM319, which is closely related to ATCC 13368, revealed a novel P450, CYP106A1 (showing 63% sequence identity towards CYP106A2), several ferredoxins as well as one flavodoxin, which support the activity of CYP106A1, it was possible to present the first cloned natural redox partners of P450s from *B. megaterium*^17^. Nevertheless, the widely used system to work with CYP106A2 in vitro as well as in vivo is the heterologous electron transfer system consisting of the soluble bovine Adx and the membrane-associated bovine AdR^5–7,13–15,18^. Adx and AdR are localized in adrenal mitochondria and realize the electron transfer to the mitochondrial cytochromes P450 CYP11A1, CYP11B1, and CYP11B2^19^. Both proteins are more or less easy to express and to purify and are able to efficiently provide electrons to CYP106A2 to convert all known substrates.

Optimization of the interaction between the redox partners and CYP106A2 could be a major advantage to conduct faster substrate conversions and to obtain higher product yields. Thus, sets of Adx mutants have been previously constructed to modify the interaction parameters with their natural redox partners from adrenal mitochondria, CYP11A1, CYP11B1, and CYP11B2. Truncation of the C-terminus by the removal of 20 amino acids, resulting in the mutant Adx(4–108), was able to accelerate the first step of the electron transfer to CYP11B1 up to 4.5-fold. Also a decreased *K*_m_ value and increased *V*_max_ value was displayed when converting 11-deoxycorticosterone by CYP11B1^20^. A further truncation by one amino acid to Adx(4–107), however, led to a poor expression of the protein^20^, indicating that the highly conserved proline at position 108 is essential for correct protein folding and incorporation of the [2Fe-2S] cluster, a fact that was verified by the elucidation of the crystal structure of Adx(4–108)^21^. The exchange of the serine at position 112 by tryptophan (S112W) and the cleavage of the 16 remaining C-terminal amino acids lowered the redox potential by 64 mV (−334 mV) compared to the wild type (−270 mV). Furthermore, Adx(S112W) showed increased *k*_cat_ values and lowered *K*_m_ values for the conversion of cholesterol to pregnenolone by CYP11A1^22^.

However, besides the replacement of the electron transfer protein Adx by mutants of Adx, the exchange with redox systems of other than mammalian origin may provide opportunities to modify the interactions between CYP106A2 and the redox partners. Therefore, we studied the influence of different redox systems on the progesterone conversion catalyzed by CYP106A2. Eleven different combinations of redox partners were tested for conversion and product pattern and included (besides Adx and AdR) the ferredoxins Adx(4–108), electron transfer protein 1 (Etp1)(516–618) (*Schizosaccharomyces pombe*), Fdx2 (*B. megaterium*), the flavodoxins FldA (*Escherichia coli*), and YkuN (*Bacillus subtilis*) as well as the reductases AdR homolog 1 (Arh1) (*S. pombe*), BmCPR (*B. megaterium*), and Fpr (*Escherichia coli*). These redox partners were chosen as they are well studied and characterized in detail. They serve as electron donors in vitro as well as in vivo to a range of bacterial and mammalian P450s, such as CYP106A2^23^, CYP106A1^24^, CYP105A1^25^, CYP109B1^26,27^, CYP267B1^28^, CYP264A1^29^, CYP11A1^30^, and CYP21A2^24,31^. The model substrate progesterone is a well-studied substrate of CYP106A2. Its main product is 15β-hydroxyprogesterone (15β-OH-P). Other monohydroxylated products are 6β-hydroxyprogesterone, 9α-hydroxyprogesterone, and 11α-hydroxyprogesterone, which, however, appear in low quantities^15,32^. In addition to the monohydroxylated progesterone products, polyhydroxylated progesterone products are also occurring, which lower the overall amount of monohydroxylated products.

We were able to demonstrate the strong influence of redox partner choice not only on the rate of substrate conversion but also on the product pattern of the multi-product conversion of progesterone by CYP106A2. The most promising ferredoxin to achieve a high yield of the main product 15β-OH-P, while reducing the amount of unwanted polyhydroxylated progesterone products, proved to be Etp1(516–618) from *S. pombe*. Using molecular docking and stopped-flow measurements, we were able to present a rational explanation for this phenomenon.

## Results

### Conversion of 30 min with 11 redox partner combinations

**Fig. 1:**
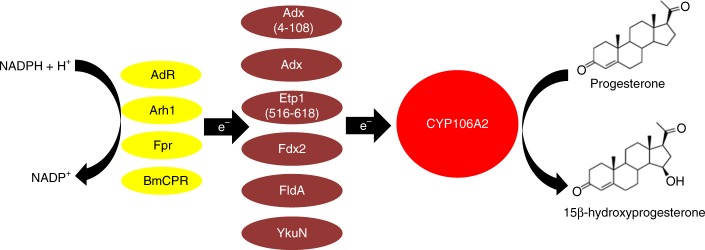
Schematic drawing of the investigated redox partner proteins. Redox equivalents are transferred from NADPH via a reductase (AdR, Arh1, Fpr, BmCPR) and a ferredoxin (Adx, Adx(4–108), Etp1(516–618), Fdx2), or flavodoxin (FldA, YkuN)) to the progesterone converting enzyme, CYP106A2

To test the ability of the various redox chains for their efficiency to transfer electrons from NAD(P)H to CYP106A2, in vitro experiments using various heterologous redox systems were conducted. The typically used redox system for CYP106A2 is the truncated form of bovine Adx, Adx(4–108), and the corresponding reductase, AdR, which was therefore used as the “parent” system. We investigated different naturally occurring redox protein systems as well as combinations between them. The redox system of *S. pombe*, consisting of the ferredoxin Etp1 and ferredoxin reductase Arh1, is completely soluble and easily expressable^33,34^. With the bacterial CYP105A1 from *Streptomyces griseolus*, the reduction efficiency was shown to be 100% for the combination of Etp1/Arh1, compared to only ~25% when using Adx(4–108)/AdR^25^. Other possibilities for reconstitution are the full-length bovine Adx, which works better with CYP11A1^35^ than the truncated form (Adx(4–108)) that is mostly used for CYP106A2, as well as redox partners from different *Bacillus* strains, such as the ferredoxin isolated from *B. megaterium* DSM319, Fdx2^[[17]]^, the typical short-chain flavodoxin YkuN from *B. subtilis*^36^, the *B. megaterium* P450 reductase BmCPR^24^, or redox partners from *E. coli*, for example, the flavodoxin FldA and NADPH-dependent flavodoxin/ferredoxin reductase Fpr^26,27,29^ (Fig. 1). These redox partners were selected as they are well studied and characterized before, and used for very different P450 species of bacterial and mammalian origin that are known to accept these redox proteins as electron donors in vitro as well as in vivo. The driving force for the ability to transfer electrons could be the redox potential (e.g., Etp1(516–618) displays a redox potential of −381 ± 2 mV^37^ compared with −344 mV^38^ for Adx(4–108)) as well as the differences in the binding mode of the redox partners to CYP106A2. Here, we focused on the interaction between both proteins and analyzed the product yield as well as the product pattern.

**Fig. 2:**
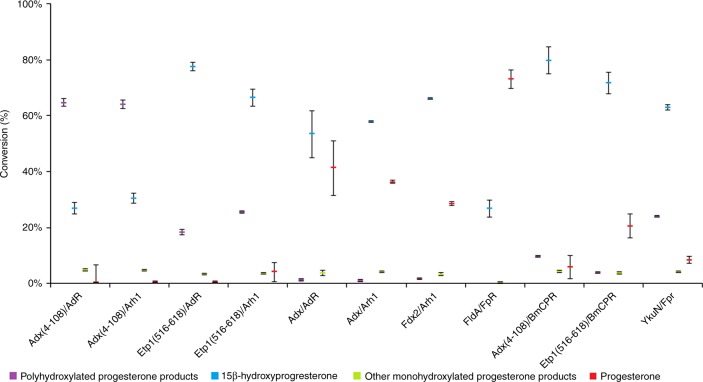
Comparison of in vitro conversions of progesterone with CYP106A2 and different redox partner combinations. Reactions were run for 30 min while keeping the ratio of P450:ferredoxin:reductase at 1:20:2 (except for P450:FldA/Fpr = 1:50:50 and P450:YkuN:Fpr = 1:10:10); violet marks indicate polyhydroxylated progesterone products, blue marks indicate the main product 15β-OH-P, green marks indicate other monohydroxylated progesterone products (e.g., 11α-hydroxyprogesterone, 9α-hydroxyprogesterone, and 6β-hydroxyprogesterone) (Fig. 3), and orange marks indicate the remaining substrate progesterone. The relative particular product level was calculated by using the relative peak area of the specific product compared to the total peak areas of reactant and products

First tests were conducted with a fixed ratio of P450:ferredoxin:reductase of 1:20:2 (exceptions for P450:FldA:Fpr = 1:50:50 and P450:YkuN:Fpr = 1:10:10) for 30 min to ensure the compatibility of redox proteins with CYP106A2 (Fig. 2).

**Fig. 3:**
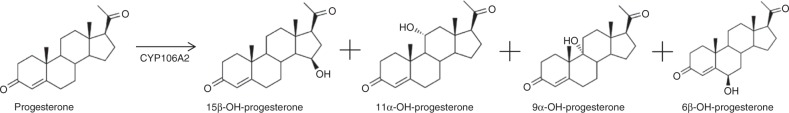
Conversion of progesterone by CYP106A2 to the monohydroxylated products, 15β-hydroxyprogesterone, 11α-hydroxyprogesterone, 9α-hydroxyprogesterone, and 6β-hydroxyprogesterone

Calculating the 15-hydroxylase activities of CYP106A2, it is obvious that the redox partner sets achieve different activities (Table 1). For example, for the use of Etp1(516–618), the activities are doubled or nearly tripled compared to those of Adx(4–108). Most interestingly, the product patterns of the progesterone conversion differ clearly between the tested combinations of redox partners (Fig. 3). Some combinations have similar overall conversion rates but produce more than 60% polyhydroxylated progesterones (e.g., Adx(4–108)/Arh1), whereas others yield more than 70% 15β-OH-P (Etp1(516–618)/AdR). Also, redox partner combinations which have slower conversion rates are distinguishable (Adx/AdR). Using the above-mentioned conditions (ratio of enzymes, temperature, time, in vitro components), it is clearly visible that the ferredoxin Adx(4–108) (combined with AdR) produces a substantial amount of polyhydroxylated products, whereas the wild-type Adx, as well as the FldA, show a lower overall conversion of progesterone (Fig. 2). Etp1(516–618) demonstrates a very high conversion (similar to Adx(4–108)/AdR) but a low amount of polyhydroxylated progesterone products. The combination of Adx(4–108) and BmCPR displays a high conversion with also low amounts of polyhydroxylated products.

**Table 1:** Calculated 15β-hydroxylase activities of CYP106A2 and progesterone

Redox proteins	15β-Hydroxylase activity of CYP106A2
Adx(4–108)/AdR	3.59
Adx(4–108)/Arh1	4.07
Etp1(516–618)/AdR	10.34
Etp1(516–618)/Arh1	8.86
Adx/AdR	7.12
Adx/Arh1	7.72
Fdx2/Arh1	8.84
FldA/FpR	3.57
Adx(4–108)/BmCPR	10.64
Etp1(516–618)/BmCPR	9.56
YkuN/Fpr	8.40

The calculated 15β-hydroxylase activities of CYP106A2 and progesterone are shown when CYP106A2 was paired with variable redox partners. Conversion time was 30 min. Activities are given in [nmol 15β-OH-P min^−1^ nmol  CYP106A2^−1^]

All combinations (except FldA/Fpr) tested did not remarkably change the amount of the other monohydroxlated progesterone products (6β-hydroxyprogesterone, 9α-hydroxyprogesterone, and 11α-hydroxyprogesterone), which always was between 3 and 5% under the conditions being tested. Switching the established bovine reductase, AdR, to the yeast Arh1 resulted in only little changes: 27% of the total product consists of the main product, 15β-OH-P, when using AdR as reductase, and 31% when using Arh1. Also, a difference in polyhydroxylated progesterone was not notable, which is 65 and 64% when using AdR and Arh1, respectively. When replacing the ferredoxin from Adx(4–108) to Etp1(516–618), the change in the product distribution is remarkable. The combination of Etp1(516–618) and AdR resulted in 78% main product and only 18% polyhydroxylated progesterone, which is a 2.6-fold increase in the main product when compared to Adx(4–108)/AdR. Also the source of reductase seems to affect the product pattern. The use of Arh1 resulted in 66% main product and 26% poly-OH-progesterone compared to 78 and 18% when AdR was used (Fig. 2). Applying the full-length Adx(1–128) completely changes the product formation: there are still 41 and 36% of the substrate left (in combination with AdR and Arh1, respectively), but there are also 53 and 58% of 15β-OH-P formed. The amount of polyhydroxylated products is negligible. The use of Fdx2 from *B. megaterium* resulted in 66% main product and 29% of unconverted substrate. No polyhydroxylated products were formed. In contrast, the redox partners originating from *E. coli*, FldA and Fpr, were not able to provide electrons to CYP106A2 when a ratio of P450:ferredoxin:reductase of 1:20:2 was applied, which resulted in no product formation. Therefore, the ratio was increased to 1:50:50, which led to a conversion of 27% (all main product). Exchanging the FldA with the YkuN from *B. subtilis* (at a ratio of 1:10:10), a production of 24% poly-OH-progesterone and 63% main product was observed. Surprisingly, the use of the very recently cloned and characterized *B. megaterium* reductase BmCPR^24^ resulted in 80 and 72% 15β-OH-P, when combined with Adx(4–108) and Etp1(516–618), and only 10 and 4% poly-OH-P formation, respectively. These data demonstrate that the application of different redox systems can alter not only the conversion rate of the substrate but also the product distribution.

### Time-course measurements for four redox partner combinations

**Fig. 4:**
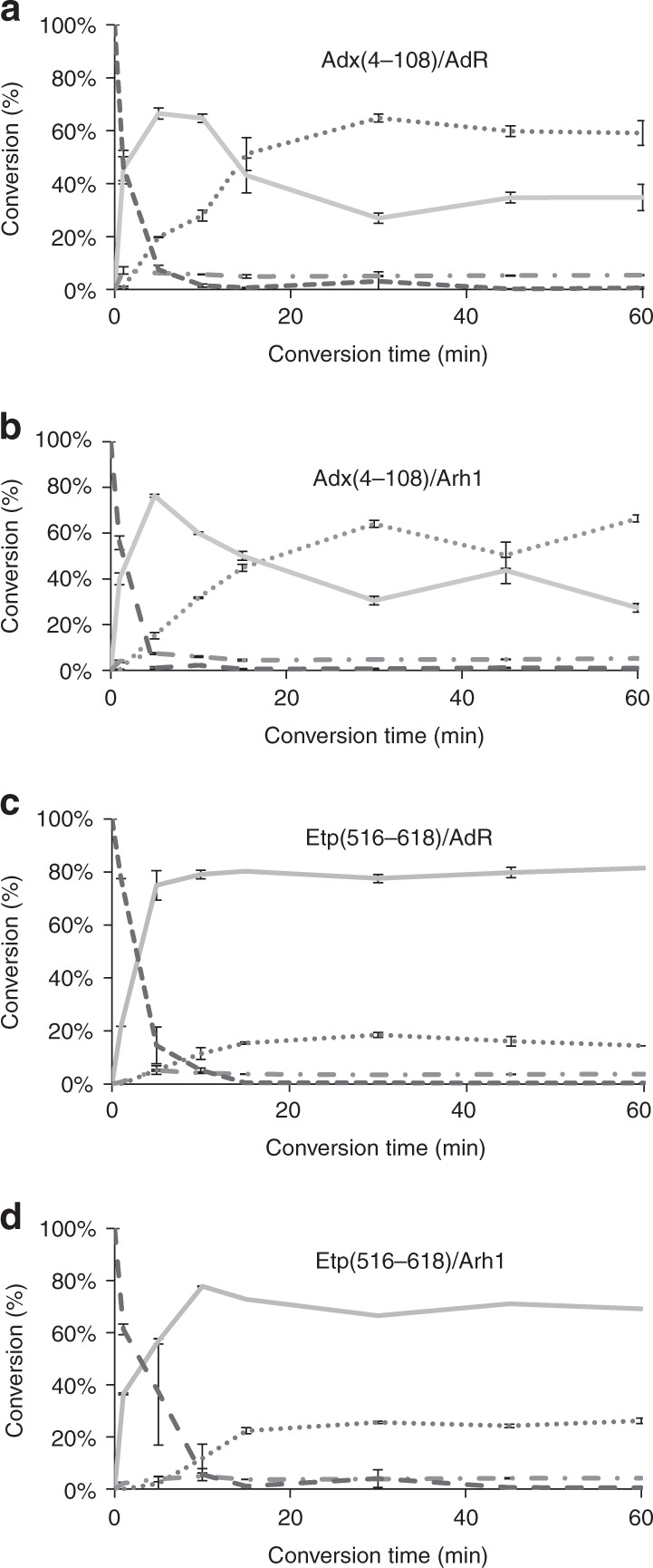
Time-dependent in vitro conversion of progesterone by CYP106A2 and different redox partner combinations. **a** Adx(4–108)/AdR, **b** Adx(4–108)/Arh1, **c** Etp1(516–618)/AdR, or **d** Etp1(516–618)/Arh1. Conversions were stopped after 1, 5, 10, 15, 30, 45, or 60 min; dotted lines indicate polyhydroxylated progesterone products, gray lines indicate the main product 15β-OH-P, dashed with single dot lines indicate other monohydroxylated progesterone products, and dashed lines indicate the remaining substrate progesterone

To gain deeper insight into the time dependence of the progesterone conversion and product distribution by CYP106A2, the combinations of the ferredoxins Adx(4–108) and Etp1(516–618) with AdR and Arh1 were investigated in more detail. When using Adx(4–108)/AdR (Fig. 4), the steep increase of 15β-OH-P formation up to 67% (5 min) is followed by a decrease to 34% after 60 min. At the same time, the amount of polyhydroxylated progesterone is slowly increasing from 7% after 5 min to 59% after 60 min. This suggests that the main product is partially further hydroxylated to result in polyhydroxylated end products. Within 10 min, 93% of progesterone has been converted.

After changing AdR for Arh1, the time dependence of progesterone conversion was mostly identical. The conversion of progesterone is almost complete after 5 min (1% left), whereas the rate of 15β-OH-P (76%) decreases rapidly after 5 min while increasing amounts of polyhydroxylated progesterones are formed (up to 66%). Using Etp1(516–618) as a ferredoxin with AdR as corresponding reductase, a steep increase of 15β-OH-P formation to 75% within in the first 5 min is followed by a slow increase up to 81% after 60 min. In this case, a slow rise of poly-OH-progesterone (not reducing the amount of the main product) to 14% after 60 min occurs. Within 10 min of conversion, 95% of the progesterone is converted. Changing the reductase for Arh1, the highest main product formation is observed after 10 min (78%), dropping slightly to 69% after 60 min. This is accompanied by an increase of poly-OH-P from 6% after 10 min to 26% after 60 min. Progesterone conversion is almost complete after 15 min (1% left). In all investigated cases, further monohydroxylated products remain at about 4% over the time-course of 60 min.

### Protein–protein docking and mutagenesis analysis

**Fig. 5:**
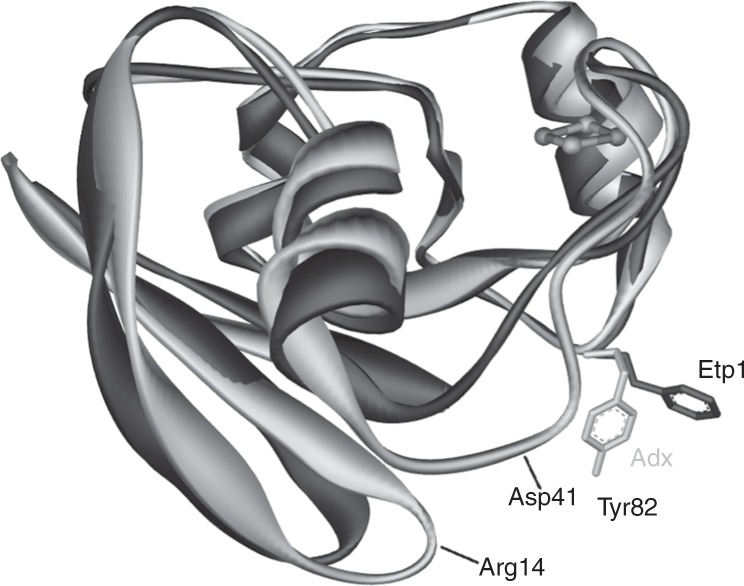
Superimposition of Adx(4–108) to Etp1(516–618). Superimposition of Adx(4–108) to the obtained docking conformation of Etp1(516–618) (dark gray), which has its FeS cluster in closest distance to CYP106A2. The loops of Adx (light gray) around residues 14 and 41 would clash with the cytochrome (omitted for clarity)

**Fig. 6:**
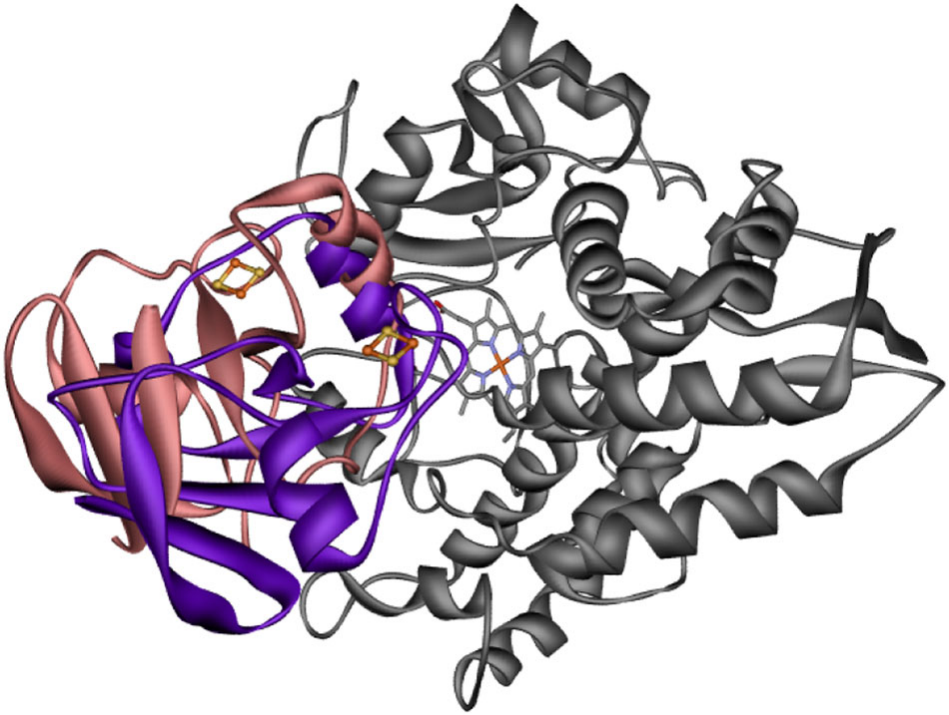
Overlay of the docking conformations of ferredoxins and CYP. Overlay of the energetically most favorable docking conformations of Adx(4–108) and of Etp1(516–618) to CYP106A2. Adx (coral) is not able to bind in the same orientation as Etp1(516–618) (violet) due to subtle but distinct structural differences

**Fig. 7:**
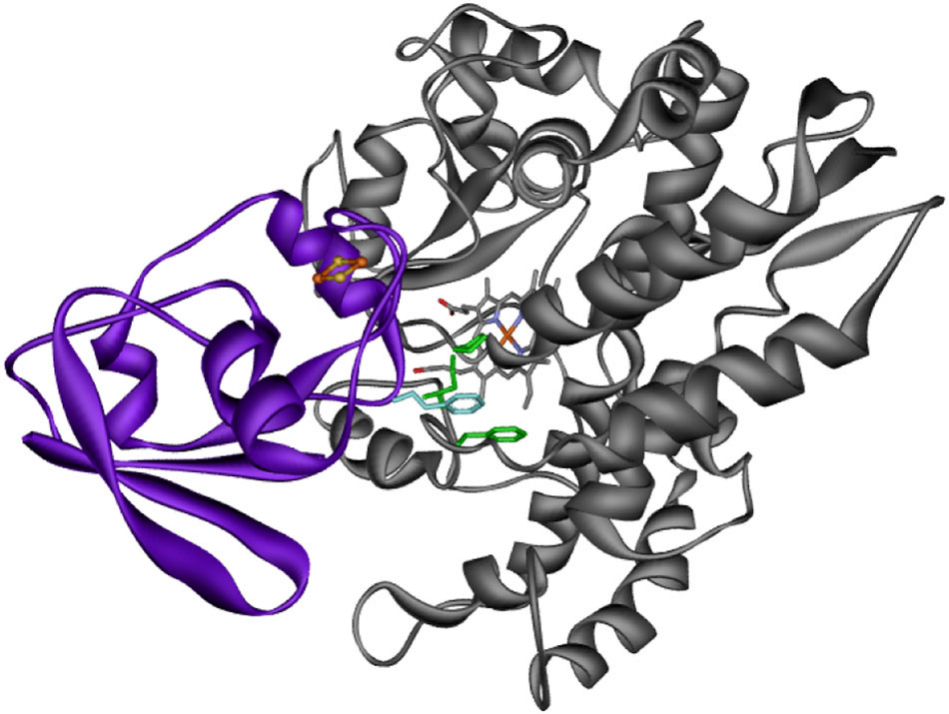
Docking conformation of Etp1(516–618) and CYP106A2. Obtained docking conformation of Etp1(516–618) (violet) in which its FeS cluster is closest to the heme group of CYP106A2 (gray). Phenylalanine (cyan) penetrates into the cytochrome forming hydrophobic contacts with Phe107, Pro359, and Leu356 (green)

Etp1(516–618) from *S. pombe*, which exhibits 81% sequence identity to the shortened, yet fully functional Adx(4–108), is also highly similar in its three-dimensional structure as the comparison of the high-resolution crystal structures shows (Fig. 5). Particularly in the region around the iron-sulfur (FeS) cluster any substantial deviations of the protein backbone are absent. Our docking studies, however, showed that subtle structural differences in the Adx(4–108) prevent it from adopting the same binding orientation to the CYP106A2 as it was found for Etp1 (Fig. 6). It turned out that the residues around positions 14 and 41 of Adx would sterically clash with the cytochrome. Furthermore, Tyr82 cannot form hydrogen bonds to the cytochrome, whereas phenylalanine in the corresponding position of Etp1 forms hydrophobic contacts, whereby penetrating deeply through the cytochrome’s surface. There, residues Phe107, Pro359, and Leu356 encase the side chain of Phe82 (Fig. 7). As a consequence, the distance between the heme-iron of the cytochrome and the FeS cluster is substantially shorter in the complex with Etp1 (19 Å) than in that with Adx(4–108) (24 Å).

To confirm that F82 of Etp1(516–618) plays an important role in the interaction and binding between Etp1(516–618) and the CYP106A2, we conducted site-directed mutagenesis studies after calculating the effect of possible mutations. The mutant F82R of Etp1(516–618) was expected to have severe effects on this interaction and was, therefore, constructed, expressed, and purified. Using this mutant, a 47% loss in the overall product formation was observed compared with the wild-type Etp1(516–618). This clearly demonstrates that the position 82 is important for the interaction of Etp1(516–618) with CYP106A2 and that mutations at this site change the binding between both proteins resulting in changed turnover numbers.

The experimentally observed hydroxylation pattern of progesterone, in particular the formation of polyhydroxylated products, requires the reorientation of monohydroxylated progesterone. In order to dissect this process of reorientation from that of the electron transfer by the ferredoxins, we performed molecular dynamics simulation of 15β-OH-P in CYP106A2. The corresponding results show that reorientation of the monohydroxylated product occurs within <100 ns. These molecular movements lead to conformations in which hydroxylation of other positions (11α-hydroxyprogesterone, 9α-hydroxyprogesterone, and 6β-hydroxyprogesterone) are possible, assuming that a distance of <5 Å between the respective carbon atom and the iron of the heme is sufficiently close for reaction.

### Stopped-flow measurements

**Fig. 8:**
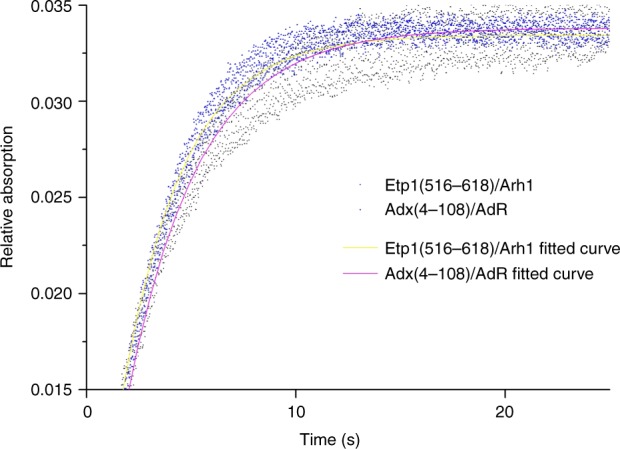
Averaged curves of stopped-flow measurement. Blue dots represent the Etp1(516–618)/Arh1 redox partner set, and black the Adx(4–108)/AdR set. Yellow and pink lines indicate the fitting, respectively. To analyze the stopped-flow reactions thoroughly, three to four replicates were averaged. The equations used were *f*(*x*) = a*(1−e^−b*x^), *b* describing the apparent rate constants (*k*_app_) for the reduction of CYP106A2 by the two redox partner combinations Adx(4–108)/AdR or Etp1(516–618)/Arh were 0.292 and 0.343 s^−1^, respectively

To validate experimentally the influence of the mode of ferredoxin binding regarding the electron transfer velocity, stopped-flow measurements were performed (Fig. 8). Differences in the reduction traces reflect the different binding behaviors that were indicated by the conversion analysis and molecular docking calculations. The resulting apparent rate constants (*k*_app_) were calculated and are presented in Table 2. Applying the redox system of Etp1(516–618)/Arh2, the apparent rate constant is 0.343 s^−1^, whereas the combination of Adx(4–108) and AdR leads to a lower *k*_app_ of 0.292 s^−1^. This is in full agreement with the calculated shorter distance of the active site of the ferredoxin Etp1(516–618) and CYP106A2 compared with Adx(4–108), as well as the faster overall conversion of progesterone when using Etp1(516–618)/Arh1.

**Table 2:** Apparent rate constants (*k*_app_) for the reduction of CYP106A2

Redox combination	*k*_app_ (s^−1^)
Adx(4–108)/AdR	0.292 ± 0.001
Etp1(516–618)/Arh	0.343 ± 0.001

Apparent rate constants (*k*_app_) for the reduction of CYP106A2 by the two redox partner combinations Adx(4–108)/AdR or Etp1(516–618)/Arh using 400 µM NADPH, 2 µM ferredoxin reductase (AdR or Arh1), and 20 µM ferredoxin (Adx(4–108) or Etp1(516–618)) in syringe A and 2 µM of CYP106A2 and 200 µM of progesterone in syringe B. Three to four replicates were averaged. The equations used to fit with the data points were: *f*(*x*) = a*(1−e^−b*x^). The given constants correspond to the respective *b* in the exponent of the equations

## Discussion

As every P450 is in need of electrons, the choice of the corresponding redox partners is crucial for the proper function of P450s^25^. A universal redox system, being able to reconstitute the activity of a majority of P450s, is the ultimate goal of many investigations^39^. There is a variety of parameters one should consider, for example, expressability, (thermo-) stability, or electron transfer rate.

In this study, we had a closer look at redox systems from different organisms that have already been used with diverse P450s. After identifying an efficient redox system for a specific P450, further optimizations by methods of protein engineering should be performed and finally tests with other P450 isoforms are designated. As a first step to create a universal redox system for P450s, we tested the applicability of diverse redox partners for the well-studied CYP106A2^6,15,40–42^. The experiments showed clearly that all used combinations of redox partners were able to provide electrons to CYP106A2, but only the truncated forms of Adx and Etp1, combined with AdR or Arh1, were able to support CYP106A2 in a way that almost all provided progesterone (200 µM) was converted within 30 min.

The most unexpected result was, however, the observation that the exchange of the redox partners resulted in a change of the product pattern. The hydroxylation of progesterone by CYP106A2 reconstituted with the commonly used redox system Adx(4–108) and AdR produces high amounts of polyhydroxylated progesterone products under the conditions studied. When using this “parent” system of Adx(4–108) and AdR for 30 min of conversion, high amounts of polyhydroxylated progesterones and low ones for 15β-OH-P are observed, a pattern that is dramatically reversed when using Etp1(516–618) (Fig. 2). Investigating the time dependency of the product formation in detail, it was shown that the conversion of progesterone by the ferredoxins is very efficient and complete within 10 to 30 min. Whereas the amount of 15β-OH-P is rising in every combination within 5 to 10 min, it is afterwards dropping substantially when using Adx(4–108). This harsh decrease is accompanied by a steep increase of poly-OH-P. In contrast, when using Etp1(516–618), the ratio of 15β-OH-P is either decreasing only slightly after 10 min or remains constant.

Based on these results, it becomes clear that the different redox partner combinations tested here can be used for diverse purposes, depending on the overall product yield and pattern one is aiming for. For approaches going for fast substrate conversions, truncated versions of ferredoxins process most of the substrate within 10 to 15 min. Adx(4–108) would be preferable to those aiming for many polyhydroxylated progesterone products which are of high interest as they represent a high diversity and, therefore, different possibilities for further alteration of the steroid molecule. Since many attempts are focused on high yields of certain monohydroxylated products, application of truncated Etp1(516–618) is useful to obtain high absolute and relative yields of the main product 15β-OH-P within only 5 min. Systems using Adx(1–128) as redox partner are a further candidate for the production of monohydroxylated progesterones because they produce very low amounts of polyhydroxylated products. However, in this case the activity is rather low.

A comparison of the ferredoxins as the direct electron transfer proteins to CYP106A2 is of special interest, because the crystal structures of the bovine Adx(4–108) and the yeast Etp1(516–618) have been solved^21,37^. Both proteins show an amino acid identity of 46% and highly conserved regions. Comparing the obtained complexes from protein–protein docking it becomes obvious that Etp(516–618) binds closer to CYP106A2 than Adx(4–108) due to steric constraints (see Figs. 5–7). This seems to be caused especially by the presence of F82 of Etp1(516–618). To confirm this hypothesis, we analyzed the effects of potential mutants at this site using computer modeling and examined experimentally the mutant F82R, proposed to have a severe effect on the interaction with CYP106A2. Compared to the wild type, the mutant showed a 47% loss in overall product formation, supporting the proposed importance of the position 82 in the interaction process. The distance between the FeS cluster of the ferredoxins (Adx(4–108) and Etp1(516–618)) and the heme-iron atom is longer (24 Å) for the binding of the Adx(4–108) than for Etp1(516–618) (19 Å). Thus, the closer approach of Etp1(516–618) should allow faster electron transfer. In fact, experimental investigation using the stopped-flow technique to study the electron transfer, revealed differences between the yeast and the adrenal redox system: the apparent rate constant (*k*_app_) for the Etp1(516–618)/Arh1 combination (yeast) is 0.343 s^−1^, while Adx(4–108)/AdR (adrenal) reaches only 0.292 s^−1^. On the other hand, the binding of Adx(4–108) might be stronger than that of Etp1(516–618), for example, due to larger electrostatic contributions, resulting in a slower release of Adx(4–108). This may lead to a longer disposition of the substrate in the active site of the CYP106A2 and, therefore, also more than one single hydroxylation product. This can be caused by the kind of contact between the ferredoxin and CYP106A2, meaning an intense binding and slow transfer of electrons will not allow the then-build product to exit the active site of CYP106A2, whereas a looser binding and fast electron transfer is enabling the monohydroxylated progesterone to leave the binding pocket before further hydroxylation is performed. In the absence of experimental data on the actual life time of these transient complexes between the respective ferredoxins and CYP106A2, corresponding assumptions must, however, be considered with some care. Nevertheless, our molecular dynamic simulations of 15β-OH-progesterone in the binding pocket of CYP106A2 show that reorientation of the monohydroxylated product occurs within 100 ns, leading to conformations in which hydroxylation of other positions (11α-hydroxyprogesterone, 9α-hydroxyprogesterone, and 6β-hydroxyprogesterone) is likely. This time scale is rather fast compared to that of the product release, which was not observed within the simulation time of 200 ns. Thus, the observed product spectra are governed by several factors, which are not independent of each other.

In summary, our results demonstrate that different aspects play a role when a decision has to be undertaken which redox system is the most preferable one for a certain P450. We were able to show that the use of different redox systems manipulates the rate and the yield of conversion of progesterone by CYP106A2. In addition, the product pattern can be altered between high levels of polyhydroxylated or monohydroxylated progesterone products by changing the redox partners. The application of Etp1(516–618) results in a high quantity of the main monohydroxylated product, whereas Adx(4–108) produces many polyhydroxylated progesterone products. Working with the full-length Adx(1–128), high amounts of monohydroxylated progesterone products are obtained with the disadvantage of slow conversion. These experimental data were supported by molecular docking revealing a shorter distance between the FeS cluster of the ferredoxin and the P450 heme for Etp1(516–618) than for Adx(4–108). Backing the molecular docking data, it was shown by stopped-flow measurements that the apparent rate constant (*k*_app_) of Etp1(516–618)/Arh1 was higher than that of Adx(4–108)/AdR, therefore resulting in a faster electron transfer between Etp1(516–618) and CYP106A2 than between Adx(4–108) and CYP106A2, explaining the above-mentioned product pattern in conversions with progesterone. Assuming a longer life time of the complex Adx(4–108) with CYP106A2 than Etp1(516–618) with CYP106A2, the monohydroxylated products are able to reorient more often applying Adx(4–108) resulting in more hydroxylations and, therefore, higher amounts of polyhydroxylated products. This knowledge can be employed to modify or tune the amount of the given products. Thus, applying the studied redox systems from bacterial, fungal, and mammalian origin, various effects on the product patterns have been observed and are promising for tuning biotechnological reactions for different purposes.

## Methods

### Site-directed mutagenesis

The Etp1(516–618) mutant was constructed in the expression vector pTrc99A. The primers (forward primer: GATTTAGCACGTGGTCTCGAGGAAACAAGCAG-3 and reverse primer: CTCGAGACCACGTGCTAAATCAAGCATGTCTTCTTC) used for the site-directed mutagenesis have been synthesized by Eurofins MWG Operon. The primers were used to construct the mutant Etp1(516–618)F82R by standard protocols^43^.

### Protein expression in *E. coli* and purification

CYP106A2, Etp1(518–618), and Arh1_A18G were expressed and purified as described elsewhere^5,7,15,33,44,45^. Arh1_A18G was used, as it demonstrates a stronger binding of the flavin adenine dinucleotide than the wild-type Arh1^33^ (further mentions of Arh1 always refer to the Arh1_A18G mutant). Adx(1–128), Adx(4–108), and AdR were purified as described before^35,38,46,47^. BmCPR was provided by Mohammed Milhim^24^. Fdx2 was provided by Elisa Brill^17^. FldA, YkuN, and Fpr were provided by Patrick Bakkes and Vlada Urlacher (University of Düsseldorf).

### In vitro conversion

A model conversion was performed in potassium phosphate buffer (50 mM, pH 7.4, 0.05% Tween-20) in a total volume of 250 µL with 0.5 µM CYP106A2, 10 µM ferredoxin or flavodoxin, and 1 µM ferredoxin reductase, resulting in a 1:20:2 ratio (P450:Fdx:FdR)^48^. As ferredoxins or flavodoxins, different varieties were employed: bovine Adx, wild-type form (amino acids 1–128, Adx(1–128)) and truncated form (amino acids 4–108, Adx(4–108)), electron transfer protein from *S. pombe* (truncated form, amino acids 516–618, Etp1(516–618)), Fdx2 from *B. megaterium* DSM319[17], YkuN from *B. subtilis*^26^, and FldA from *E. coli*^27^. The following enzymes were applied as reductases: bovine AdR, AdR homolog 1 from *S. pombe* (Arh1), BmCPR from *B. megaterium*^24^ and Fpr from *E. coli*^27^. 200µM progesterone was used as substrate and the reaction was started by the addition of 0.1 mM NADPH. A NADPH-regenerating system containing 1 mM MgCl_2_, 5 mM glucose-6-phosphate, and 4 U mL ^−1^ glucose-6-phosphate dehydrogenase was used. The conversion took place at 30 °C and 900 rpm for 1 to 60 min; the reactions were quenched and extracted with 500 µL of ethyl acetate; the organic phases were evaporated to dryness and stored at −20 °C until high-performance liquid chromatography (HPLC) analysis. Three samples were conducted for each tested combination of redox partners.

### HPLC analysis

Reversed-phase liquid chromatography was performed using a Jasco system containing an UV-2075Plus Intelligent UV/Vis Detector, an AS-2050Plus Intelligent Sampler, an LG-2080-02 Ternary Gradient Unit, a PU-2080Plus Intelligent HPLC Pump, a DG-2080-53 3-Line Degasser, and an LC-NetII/ADC (Jasco, Gross-Umstadt, Germany). The mobile phase acetonitrile/water 10:90 was degassed prior to use and the flow rate was 1 mL min^−1^ with the following gradient: a linear gradient for 2 min from 89% solvent A (water containing 10% acetonitrile) to 33% solvent B (100% acetonitrile) holding it for 4 min, a linear gradient for 4 min to 100% solvent B holding it for 2 min, a linear gradient for 0.1 min to 89% solvent A holding it for 3 min. A reversed-phase ec MN Nucleodor C18 (5 µm, 4.0 × 125 mm^2^) column (Macherey-Nagel, Düren, Germany) was adjusted to 40 °C by a column thermostat (W.O. Electronics, Austria). For each measurement, 10 µL of a sample was injected and the absorbance was monitored at 240 nm. The relative product level was evaluated using the relative peak area of the product compared to the combined peak areas of educt and product. The values of the three samples were averaged out (mean average), and standard deviations were calculated.

### Computational methods

The homology models of the Adx mutants where constructed applying the corresponding functions of the Swiss-PdbViewer (version 4.01, http://www.expasy.org/spdbv) to the crystal structure of bovine Adx (PDB entry code 1AYF, chain B), which comprises residues 5–108^21^. The Adx-like Etp1 was prepared likewise (PDB entry code 2WLB, chain B)^37^. As receptor, the conformation of CYP106A2 from *B. megaterium* in substrate-bound form was used (PDB entry code 5IKI, chain B). For protein–protein docking the ClusPro2 web service (https://cluspro.org/login.php) was used, applying default parameters^49–52^. Due to the limitations of the underlying method all non protein parts (heme group of the cytochrome as well as the Fe_2_S_2_ fragment of Adx and of etp1) were removed prior to the docking procedure. For further analysis the complexes obtained according to the „balanced scoring function“ were used. These were inspected manually by superimposing the heme containing structure of CYP106A2 within vmd (version 1.8.6, http://www.ks.uiuc.edu/Research/vmd/)^53^.

Molecular dynamics simulations were carried out on 15β-OH-progesterone in the binding pocket of CYP106A2 applying GROMACS (version 4.4.5)^54^. The amino acids that were unresolved in the crystal structure (residues 176 to 180) were modeled using the Swiss-PdbViewer. The cytochrome was placed in the center of a cubic box with a minimum distance of 10 Å from the box edge and surrounded by TIP3P waters^55^. Energy minimization was started from the putative conformation of on 15β-OH-progesterone after hydroxylation. The steepest descent energy minimization was stopped at a gradient norm below 1000 kJ mol^−1^ nm^−2^. Equilibration consisted in two different steps: first in a NVT (constant volume, temperature, and number of particles) ensemble for 100 ps maintaining the temperature at 300 K using a modified Berendsen thermostat^56^, and second by stabilizing the system at 1 bar applying a Parinello-Rahman barostat^57^ for 100 ps of NPT (constant pressure, temperature, and number of particles) equilibration. All bonds in both steps were constrained by the LINCS algorithm^58^. Subsequently the production run of 200 ns was performed. Hereby Newton’s equations of motion were integrated by the leapfrog algorithm at time steps of 2 fs. Snapshots of the trajectory were taken at intervals of 1 ps.

### Stopped-flow measurements (kinetics by rapid mixing)

Stopped-flow measurements were carried out on a SFM 300 stopped-flow spectrophotometer equipped with an FC-15 cuvette and an MPS 60 data-processing unit (Biologic SAS, Claix, France) at 20 °C, as described previously^22,59^. The system was made anaerobic by repeated flushing with excessively argon-bubbled buffer. The reaction buffer for CYP106A2 consisted of 50 mM HEPES (pH 7.4) and 0.05% Tween-20. Conditions were optimized for the reduction of CYP106A2. All samples were prepared in a glove box in an oxygen-free atmosphere. To follow the whole cascade of reactions within one measurement (i.e., reduction of ferredoxin reductase, ferredoxin, and CYP106A2), syringe A contained 400 µM NADPH, 2 µM ferredoxin reductase (AdR or Arh1), and 20 µM ferredoxin (Adx(4–108) or Etp1(516–618)) and syringe B contained 2 µM CYP106A2 and 200 µM progesterone. The mixture in syringe A was allowed to age for at least 15 min to allow the binding of the different components. The solution in syringe B was CO saturated prior to loading into the driving syringe.

The reaction was monitored via the CO complex formation showing an absorbance maximum at 450 nm and representing the formation of the reduced species. In total 6001 data points were recorded with a split time of 10/50 s. The dead time for the measurements with the SX-17MV was 2.6 ms. For each combination of redox partners three to four replicates traces were averaged. All resulting curves were evaluated using Origin. The reduction of CYP106A2 by ferredoxin species Adx(4–108) or Etp1(516–618) is best described by monoexponential relaxations, which were fitted to the data resulting in the apparent rate constants (*k*_app_).

### Data availability

The datasets generated and analyzed during the current study are available from the corresponding author on reasonable request. The following previously published protein structures were used in this study: bovine Adx (PDB entry code 1AYF, chain B); adrenodoxin-like Etp1 (PDB entry code 2WLB, chain B); and CYP106A2 from *Bacillus megaterium* in substrate-bound form (PDB entry code 5IKI, chain B).
